# The effect of gonadotropin-releasing hormone agonist downregulation in conjunction with hormone replacement therapy on endometrial preparation in patients for frozen–thawed embryo transfer

**DOI:** 10.3389/fmed.2024.1412126

**Published:** 2024-07-03

**Authors:** Haoying Hao, Meng Li, Cuilian Zhang, Shaodi Zhang

**Affiliations:** Reproductive Medicine Center, Henan Provincial People’s Hospital, People’s Hospital of Zhengzhou University, Zhengzhou, China

**Keywords:** frozen–thawed embryo transfer, GnRHa, HRT, leuprorelin, clinical outcomes

## Abstract

**Objective:**

To investigate the effects of combining gonadotropin-releasing hormone agonist (GnRHa) downregulation with hormone replacement therapy (HRT, GnRHa-HRT) on the clinical outcomes of patients undergoing frozen–thawed embryo transfer (FET).

**Methods:**

In this retrospective study, we included patients who had FET between January 2018 and December 2022. They were categorized into HRT and GnRHa-HRT groups based on the endometrial preparation protocol. The study compared the clinical outcomes of patients in two groups. Possible factors affecting clinical outcomes were analyzed using univariate analysis. To analyze the impact of two endometrial preparation methods on clinical outcomes, multifactorial logistic regression was performed.

**Results:**

The rates of clinical pregnancy (47.31% vs. 59.60%), embryo implantation (37.58% vs. 49.65%), biochemical pregnancy (52.36% vs. 64.31%), and early abortion (7.07% vs. 10.77%) were statistically different between the two groups (*p* < 0.05). Analysis using multifactorial logistic regression showed that there was a 1.65-fold increase in clinical pregnancy rates (OR = 1.65, 95% CI: 1.29–2.12, *p* < 0.001) and a 1.55-fold increase in embryo implantation rates (OR = 1.55, 95% CI: 1.27–1.90, *p* < 0.001) in the GnRHa-HRT group when compared to the HRT group. For blastocyst transfer, the clinical pregnancy and implantation rates of the GnRHa-HRT group were significantly higher than those of the HRT group (OR = 1.75, 95% CI: 1.30–2.37, *p* < 0.001; OR = 1.73, 95% CI: 1.35–2.21, *p* < 0.001).

**Conclusion:**

In FET cycles, leuprorelin (as a GnRHa) downregulation combined with HRT may improve the clinical outcome of patients compared to the HRT cycle, especially for the clinical pregnancy and embryo implantation rates of patients with blastocyst transfer.

## 1 Introduction

Frozen–thawed embryo transfer (FET) has steadily grown in importance as a supplementary technique in the advancement of human-assisted reproductive technology due to its simplicity of operation and high safety features (1). Endometrial preparation is a critical stage of the FET cycle, which is crucial to the success of embryo implantation and influences the pregnancy outcomes following the transfer. It is available in several protocols, including natural, ovulation promotion, hormone replacement therapy (HRT), and downregulation of the HRT cycles (2).

For different endometrial preparation protocols, the natural cycle is simple, economical, and suitable for patients with normal ovulation. Patients with irregular menstruation and ovulation disorders are suitable for the ovulation promotion cycle. The HRT cycle is suitable for patients who need to cancel the natural cycle or the ovulation induction cycle for various reasons. Studies have shown that the HRT cycle increases the possibility of pregnancy compared to patients with natural cycles and the same results have been found in patients with a thin endometrium (3).

The combination of gonadotropin-releasing hormone agonist downregulation and HRT (GnRHa-HRT) prevents unexpected ovulation during the HRT cycle (4). In addition, GnRHa has been used for long-term pituitary suppression in FET cycles (5). The affinity of GnRHa to the receptor is much higher than gonadotropin-releasing hormone (GnRH) secreted by the hypothalamic, which can lower pituitary sensitivity and reduce or inhibit the occurrence of spontaneous luteinizing hormone (LH) surge. It not only synchronizes follicular development but also improves the receptivity of the endometrium (6). Leuprorelin, also called leuprolide, is a GnRHa that has been studied for the treatment of endometriosis, adenomyosis, and uterine fibroids (7–9). For patients with FET, uterine conditions are closely related to pregnancy, and leuprorelin is commonly used for uterine disorders or in combination with other medications (10, 11).

For the different endometrial preparation protocols commonly used, studies have shown that there is no significant difference in the HRT cycle or GnRHa-HRT cycle and the latter protocol increases the cost of treatment (12, 13). On the contrary, studies have also shown that GnRHa-HRT effectively improved clinical pregnancy (14) and live birth rates (15). Based on the results of the above studies, some controversies about the advantages and disadvantages of the HRT cycle or GnRHa-HRT cycle in endometrial preparation protocols still exist. Therefore, we retrospectively gathered clinical data from patients who underwent FET after the cancelation of fresh cycle transfer or non-pregnancy after the first embryo transfer due to variable factors such as abnormal endometrium or hormone levels. This research aimed to evaluate the effects of HRT vs. GnRHa-HRT on the clinical outcomes in patients who underwent FET.

## 2 Materials and methods

### 2.1 Study design and population

This study was a retrospective cohort analysis conducted on patients who had FET with HRT cycles between January 2018 and December 2022 at the Reproductive Medicine Center of Henan Provincial People’s Hospital. The inclusion criteria for patients were as follows: (1) patients who underwent FET after the cancelation of the fresh cycle due to their endometrium or hormone levels, or non-pregnancy after the first embryo transfer; (2) patients who received HRT or GnRHa plus HRT (downregulation + HRT, with leuprorelin). The exclusion criteria were as follows: (1) recurrent miscarriages and repeated implantation failures; (2) frozen eggs and egg recipients; (3) intrauterine adhesions, adenomyosis, uterine fibroids, endometrial polyps, and congenital uterine malformation; (4) either spouse with abnormal chromosomes; (5) presence of internal medical diseases such as diabetes and hypertension. This research was granted ethical approval by the Ethics Committee of Henan Provincial People’s Hospital (Approval No. SYSZ-LL-2019110401). Prior to treatment, all patients provided informed consent. Patients were categorized into the HRT group and the GnRHa-HRT group.

### 2.2 Embryo cryopreservation and thawing

Blastocyst embryos were graded based on the Gardner and Schoolcraft criteria (16), and cleavage-stage embryos were evaluated according to the scoring system as described by Dale et al. (17). All embryos were preserved by vitrification (Vitrification Kit, Kitazato, Japan) and thawed when transferred.

### 2.3 Endometrial preparation protocol

HRT cycle: Patients orally took estradiol valerate (1 mg/tablet, 4–6 mg/d × 7 days) from the 2nd to 4th day of their menstrual cycle or withdrawal bleeding. After 7 days, ultrasound was used to monitor the endometrial thickness (EMT), and the dose of estradiol valerate was modified accordingly. The maximum dose was 8 mg/day, and the total time of use was 11 to 20 days. When the EMT was measured at least 8 mm or human chorionic gonadotropin (hCG) was administered in the ovulation cycle, the endometrium was transformed with progestogen. The dose of estradiol valerate was kept unchanged, and progesterone was given in the form of a vaginal slow-release gel (90 mg/capsule, 1 capsule/d) in conjunction with oral dydrogesterone tablets (10 mg/tablet, 20 mg/d). Cleavage embryos were transferred 4 days post-transformation, and blastocysts were transferred on the 6th day.

GnRHa-HRT cycle: Between the 2nd and 3rd days of the menstrual cycle, the long-acting GnRHa (leuprorelin, Beijing Biote Pharmaceutical Co., Ltd., 3.75 mg) was subcutaneously injected. When the endothelium was <5 mm and progesterone <1.0 ng/mL after 14–21 days, the estradiol valerate (8 mg/day) was given and was administered for ≥9 days. Endometrium was transformed when EMT was ≥8 mm, cleavage embryos were transferred 4 days post-transformation, and blastocysts were transferred on the 6th day. The luteal support of GnRHa-HRT was the same as HRT.

The serum β-human chorionic gonadotropin (β-hCG) levels were checked 2 weeks following the transfer of the embryo. If the result was positive, the patient would continue taking estradiol valerate and progesterone daily until a fetal heartbeat was seen on ultrasound, and the dosage would be reduced gradually and discontinued by the 10th week of pregnancy.

### 2.4 Outcome indicators

The primary outcomes included clinical pregnancy and live birth rates. Clinical pregnancy was determined using ultrasound to confirm the presence of a gestational sac 4–6 weeks after the embryo transfer. A live birth refers to the successful delivery of a living baby after 28 weeks of pregnancy. The secondary outcomes were embryo implantation, multiple pregnancy, biochemical pregnancy, and early abortion rates. Successful embryo implantation refers to the interaction between the embryo and the endometrium, and implanting into the endometrium. Multiple pregnancy refers to more than one fetus in a pregnancy. Biochemical pregnancy was characterized as β-hCG >25 IU/L in serum at 12–14 days of transplantation. Early abortion was defined as miscarriage or the cessation of embryonic growth before 12 weeks of gestation.

### 2.5 Statistical analysis

Statistical analysis was conducted using SPSS 27.0. The Shapiro–Wilk test was utilized to assess normal distribution, and non-normally distributed continuous variables were presented as medians (IQR). Group comparisons were conducted using the Wilcoxon test. Categorical variables were evaluated utilizing either the chi-square test or Fisher’s exact test. Possible factors affecting clinical outcomes were analyzed using univariate analysis. The effects of the two endometrial preparation protocols on clinical outcomes were analyzed using multifactorial logistic regression after adjusting for confounding factors. A statistical significance was determined if the *p* < 0.05.

Propensity score matching was used to match the study objects according to HRT and GnRHa-HRT protocols using 1:1 nearest neighbor matching method, and the matching tolerance was set at 0.0005. Female age, male age, endometriosis, anti-Müllerian hormone (AMH), developmental stages of transferred embryos, embryo quality, the number of embryos transferred, and EMT on the day of transfer were used as matching variables. According to the data from our center, it is assumed that the clinical pregnancy rate in the GnRHa-HRT group is 66% (p_1_), while in the HRT group it is 56% (p_2_). The hypothesis testing is conducted with a type I error (α) set at 0.05 and a type II error (β) at 0.1. The sample size ratio (k) of the two groups is 1:1. Utilizing the sample size formula: n_2_ = (z_1-α/2_ + z_1-β_)^2^ × [p_1_ (1-p_1_)/k + p_2_ (1-p_2_)]/(p_1_-p_2_)^2^, n_1_ = k × n_2_, where z_1-α/2_ = 1.96, z_1-β_ = 1.28, yielding a sample size of 495 for the GnRHa-HRT group and 495 for the HRT group.

## 3 Results

### 3.1 Baseline characteristics

**Figure 1:**
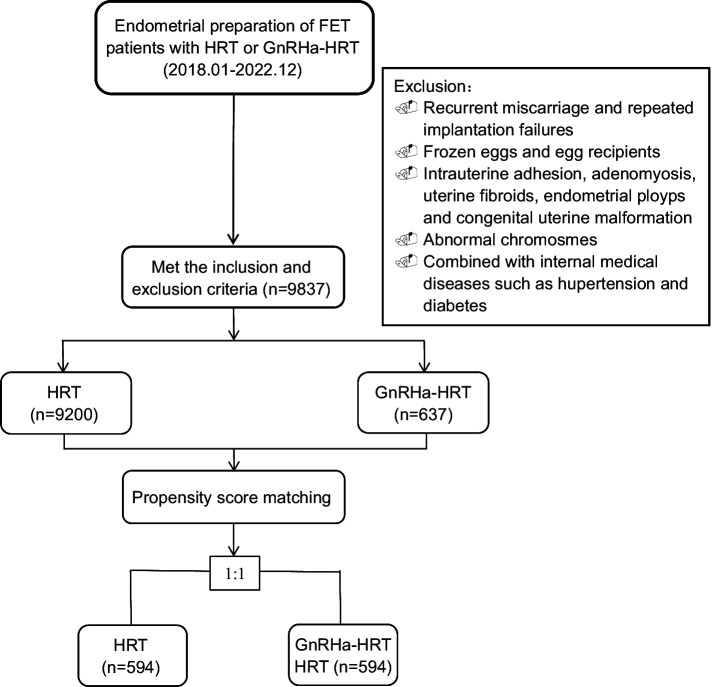
Patient inclusion flowchart. FET, frozen–thawed embryo transfer; HRT, hormone replacement therapy; GnRHa-HRT, gonadotropin-releasing hormone agonist downregulation and HRT.

There were 9,200 patients in the conventional HRT group who met the criteria and 637 patients in the GnRHa-HRT group. After PSM, there were 594 patients in each of the two groups (Figure 1). The baseline characteristics of the matched patients are shown in Table 1. Statistical differences were observed in the EMT, developmental stages of transferred embryos, and the number of embryos transferred among the two groups (*p* < 0.05). However, there were no significant differences in male factors, female age, female BMI, duration of infertility, presence of endometriosis, infertility types, AMH, fertilization mode, and quality of transferred embryos.

**Table 1:** Baseline characteristics of patients with HRT cycle and GnRHa-HRT cycle [Mean (SD) Median (Q1-Q3) / *N* (%)].

Variable	HRT (*n* = 594)	GnRHa-HRT (*n* = 594)	*p*-value
Age, years	31 (28, 36)	31 (29, 35)	0.547
BMI, kg/m^2^	23 (20.8, 25.4)	22.58 (20.69, 25.28)	0.136
Male factors			
Age, year	32 (28, 37)	32 (29, 35)	0.853
BMI, kg/m^2^	25.08 (22.86, 27.51)	25.25 (22.86, 27.68)	0.737
Endometriosis, *n* (%)			0.560
Yes	37 (6.23%)	42 (7.07%)	
No	557 (93.77%)	552 (92.93%)	
Duration of infertility, years	3 (2, 5)	3 (1.5, 5)	0.123
Infertile type, *n* (%)			0.680
Secondary infertility	343 (57.74%)	350 (58.92%)	
Primary infertility	251 (42.26%)	244 (41.08%)	
AMH, ng/ml	3.36 (1.64, 5.54)	3.5 (1.85, 5.7)	0.244
EMT, cm	9.6 (8.8, 10.78)	10 (9, 11)	0.009
Mode of fertilization, *n* (%)			0.787
IVF	448 (75.42%)	452 (76.09%)	
ICSI	146 (24.58%)	142 (23.91%)	
Type of embryo transferred, *n* (%)			0.004
Cleavage-stage embryo	207 (34.85%)	161 (27.1%)	
Blastocyst-stage embryo	387 (65.15%)	433 (72.9%)	
No. of embryos transferred			<0.001
1	270 (45.45%)	334 (56.23%)	
2	324 (54.55%)	260 (43.77%)	
Embryo quality, *n* (%)			0.448
High	334 (56.23%)	321 (54.04%)	
Not high	260 (43.77%)	273 (45.96%)	

HRT, hormone replacement therapy; GnRHa-HRT, gonadotropin-releasing hormone agonist downregulation and HRT; BMI, body mass index; AMH, anti-Müllerian hormone; EMT, endometrial thickness; IVF, in vitro fertilization; ICSI, intracytoplasmic sperm injection.

### 3.2 Clinical outcomes

There were significant differences in the rates of clinical pregnancy (47.31% vs. 59.60%), embryo implantation (37.58% vs. 49.65%), biochemical pregnancy (52.36% vs. 64.31%), and early abortion rates (7.07% vs. 10.77%) between HRT and GnRHa-HRT groups (*p* < 0.05). The differences in the rates of live birth (38.55% vs. 43.10%) and multiple pregnancies (10.94% vs. 12.29%) did not show statistical significance (*p* > 0.05). Furthermore, there were no ectopic pregnancies in the HRT group and two in the GnRHa-HRT group (Table 2).

**Table 2:** Clinical outcomes of patients with HRT cycle and GnRHa-HRT cycle.

Variable	HRT (*n* = 594)	GnRHa-HRT (*n* = 594)	*P*-value
Primary outcome indicators			
Clinical pregnancy, *n* (%)			<0.001
Yes	281 (47.31%)	354 (59.60%)	
No	313 (52.69%)	240 (40.40%)	
Live birth rate			0.111
Yes	229 (38.55%)	256 (43.10%)	
No	365 (61.45%)	338 (56.90%)	
Secondary outcome indicators			
Embryo implantation, *n* (%)			<0.001
Yes	345 (37.58%)	424 (49.65%)	
No	573 (62.42%)	430 (50.35%)	
Biochemical pregnancy, *n* (%)			<0.001
Yes	311 (52.36%)	382 (64.31%)	
No	283 (47.64%)	212 (35.69%)	
Multiple pregnancy, *n* (%)			0.469
Yes	65 (10.94%)	73 (12.29%)	
No	529 (89.06%)	521 (87.71%)	
Early abortion, *n* (%)			0.025
Yes	42 (7.07%)	64 (10.77%)	
No	552 (92.93%)	530 (89.23%)	

### 3.3 Univariate analysis

According to the univariate analysis, male age, female age, duration of infertility, AMH, endometrial preparation protocols, embryo development stage, embryo quality, EMT on the transplantation date, and the presence of endometriosis may influence the clinical pregnancy (*p* < 0.05). Male age, female age, AMH, embryo development stage, embryo quality, and EMT may influence the live birth rate (*p* < 0.05). In addition, male age, female BMI, female age, duration of infertility, AMH, endometrial preparation protocols, embryonic development stage, number of transplanted embryos, EMT on the transplantation date, and the presence of endometriosis may influence embryo implantation (*p* < 0.05) (Table 3).

**Table 3:** Univariate analysis affecting embryo implantation rate, clinical pregnancy rate, and live birth rate in thawing cycles.

Variable	Clinical pregnancy rate		Live birth rate		Embryo implantation rate	
	OR (95% CI)	*P*-value	OR (95% CI)	*P*-value	OR (95% CI)	*P*-value
Age	0.91 (0.89, 0.93)	<0.001	0.90 (0.88, 0.92)	<0.001	0.91 (0.89, 0.93)	<0.001
BMI	0.97 (0.94, 1.01)	0.112	0.98 (0.95, 1.02)	0.32	0.97 (0.94, 1.00)	0.026
**Male factors**						
Male age	0.93 (0.91, 0.95)	<0.001	0.92 (0.90, 0.94)	<0.001	0.93 (0.91, 0.95)	<0.001
Male BMI	1 (0.97, 1.03)	0.993	1.01 (0.98, 1.04)	0.690	0.99 (0.96, 1.01)	0.290
Duration of infertility	0.95 (0.92, 0.99)	0.010	0.97 (0.93, 1.00)	0.084	0.94 (0.91, 0.97)	<0.001
**Infertile type**						
Secondary infertility	Ref		Ref		Ref	
Primary infertility	0.86 (0.68, 1.09)	0.212	0.88 (0.69, 1.11)	0.277	0.84 (0.7, 1.02)	0.083
AMH	1.09 (1.05, 1.12)	<0.001	1.08 (1.04, 1.11)	<0.001	1.08 (1.05, 1.12)	<0.001
**Endometrial preparation protocols**						
HRT	Ref		Ref		Ref	
GnRHa-HRT	1.64 (1.31, 2.07)	<0.001	1.21 (0.96, 1.52)	0.111	1.64 (1.36, 1.98)	<0.001
Developmental stages of transferred embryos						
Cleavage-stage embryo	Ref		Ref		Ref	
Blastocyst-stage embryo	1.55 (1.21, 1.98)	0.001	1.33 (1.03, 1.71)	0.028	1.9 (1.56, 2.32)	<0.001
**Number of embryos transferred**						
1	Ref		Ref		Ref	
2	1.19 (0.95, 1.5)	0.135	1.26 (1.00, 1.59)	0.050	0.61 (0.5, 0.75)	<0.001
**Embryo quality**						
High	Ref		Ref		Ref	
Not high	0.73 (0.58, 0.92)	0.007	0.73 (0.57, 0.92)	0.007	0.87 (0.72, 1.05)	0.157
EMT	1.2 (1.12, 1.29)	<0.001	1.18 (1.10, 1.26)	<0.001	1.18 (1.12, 1.25)	<0.001
**Mode of fertilization**						
IVF	Ref		Ref		Ref	
ICSI	1.14 (0.87, 1.49)	0.338	1.11 (0.85, 1.45)	0.455	1.14 (0.92, 1.42)	0.230
**Endometriosis**						
Yes	Ref		Ref		Ref	
No	0.61 (0.38, 0.98)	0.042	0.73 (0.46, 1.15)	0.175	0.69 (0.47, 1)	0.050

BMI, body mass index; AMH, anti-Müllerian hormone; HRT, hormone replacement therapy; GnRHa-HRT, gonadotropin-releasing hormone agonist downregulation and HRT; EMT, endometrial thickness; IVF, in vitro fertilization; ICSI, intracytoplasmic sperm injection.

### 3.4 Multifactorial logistic regression analysis

In the adjusted model, the results revealed that in the GnRHa-HRT group, the clinical pregnancy and embryo implantation rates were 0.55 times higher (OR = 1.55, 95% CI: 1.27–1.90, *p* < 0.001) and 0.65 times higher (OR = 1.65, 95% CI: 1.29–2.12, *p* < 0.001) than those in the HRT group, respectively. However, there was no significant difference in the live birth rate (OR = 1.21, 95%CI:0.93–1.52, *p* = 0.175) (Table 4).

**Table 4:** Multifactorial logistic regression analysis of the effects of different endometrial preparation protocols on embryo implantation rate, clinical pregnancy rate, and live birth rate.

Variable	Clinical pregnancy rate				Live birth rate				Embryo implantation rate			
	Unadjusted		Adjusted		Unadjusted		Adjusted		Unadjusted		Adjusted	
	OR (95% CI)	*P*-value	OR (95% CI)	*P*-value	OR (95% CI)	*P*-value	OR (95% CI)	*P*-value	OR (95% CI)	*P*-value	OR (95% CI)	*P*-value
HRT	Ref		Ref		Ref		Ref		Ref		Ref	
GnRHa-HRT	1.64 (1.31, 2.07)	<0.001	1.65 (1.29, 2.12)	<0.001	1.21 (0.96, 1.52)	0.111	1.19 (0.93, 1.52)	0.175	1.64 (1.36, 1.98)	<0.001	1.55 (1.27, 1.90)	<0.001

^a^Adjustment: male/female age, male/female BMI, endometriosis, AMH, developmental stages of transferred, number of embryos transferred, EMT, duration of infertility, infertility type, and fertilization method.

Furthermore, the embryo implantation and clinical pregnancy rates were also significantly higher with GnRHa-HRT in the group of patients who were transferred blastocyst-stage embryos (OR = 1.73, 95% CI: 1.35–2.21, *p* < 0.001; OR = 1.75, 95% CI: 1.30–2.37, *p* < 0.001). However, in the group of patients transferred with cleavage-stage embryos, there was no significant difference (OR = 1.18, 95% CI: 0.72–1.91, *p* = 0.513; OR = 1.07, 95% CI: 0.73–1.56, *p* = 0.728). Meanwhile, in the groups of blastocyst-stage embryos transferred and cleavage-stage embryos transferred, there were no significant differences in the live birth rate between HRT and GnRHa-HRT (Table 5).

**Table 5:** Multifactorial logistic regression analysis of the effects of endometrial preparation protocols on embryo implantation rate, clinical pregnancy rate, and live birth rate of different developmental stages of embryos transferred.

Variable	Clinical pregnancy rate				Live birth rate				Embryo implantation rate			
	Unadjusted		Adjusted		Unadjusted		Adjusted		Unadjusted		Adjusted	
	OR (95%CI)	*P*-value	OR (95%CI)	*p*-value	OR (95%CI)	*p*-value	OR (95%CI)	*p*-value	OR (95%CI)	*p*-value	OR (95%CI)	*p*-value
**Cleavage-stage group**												
HRT	Ref		Ref		Ref		Ref		Ref		Ref	
GnRHa-HRT	1.43 (0.95,2.17)	0.09	1.18 (0.72,1.91)	0.513	1.20 (0.78, 1.84)	0.404	1.14 (0.69, 1.90)	0.609	1.33 (0.96,1.85)	0.09	1.07 (0.73,1.56)	0.728
**Blastocyst-stage group**												
HRT	Ref		Ref		Ref		Ref		Ref		Ref	
GnRHa-HRT	1.68 (1.27, 2.22)	<0.001	1.75 (1.30, 2.37)	<0.001	1.18 (0.89, 1.55)	0.251	1.16 (0.87, 1.56)	0.315	1.67 (1.32, 2.11)	<0.001	1.73 (1.35, 2.21)	<0.001

^a^Adjustment: male/female age, male/female BMI, endometriosis, AMH, number of embryos transferred, EMT, duration of infertility, infertility type, and fertilization method.

## 4 Discussion

The patients involved in this research were those who underwent FET following the cancelation of fresh cycle transfer or non-pregnancy after the initial embryo transfer, attributed to factors such as their endometrium or hormone levels, which is different from the inclusion scope of other studies (11, 18, 19). Endometrium factors may include thin endometrium and endometriosis. Hormone levels involve estrogen, progestin, follicle-stimulating hormone, luteinizing hormone, and androgen, and any abnormality of these factors can lead to infertility. Leuprorelin may improve the clinical symptoms of infertility patients by affecting the above hormones and is commonly used in treating endometriosis (20). Previous studies have not examined the clinical outcomes of downregulation with leuprorelin alone in the same range as included in this study.

This research included patients over a period of nearly 5 years and had relatively complete baseline data. The PSM was performed on the study population according to the HRT and GnRHa-HRT protocols using the 1:1 nearest neighbor matching method to avoid potential confounders and selection bias. The reliability of the results was therefore enhanced. EMT affects endometrial receptivity, some studies have shown better FET outcomes with the EMT > 7 mm. The rate of clinical pregnancy for an EMT of 7 mm or less is 23.3%, which is significantly lower than the 48.1% rate observed in cases where the EMT exceeds 7 mm (21). Liu et al. also found that patients with an EMT < 8 mm exhibited a heightened likelihood of giving birth to infants classified as small for gestational age (22). Therefore, the threshold for EMT on the day of transformation was set at 8 mm in this study.

Currently, studies have shown that the GnRHa-HRT cycle has a positive therapeutic effect on FET patients with thin endometrium or adenomyosis (23, 24). It can also improve the reproductive outcomes for older patients (aged 36–43 years) who in experience recurrent implantation failure in FET cycles (25). Prior treatment with GnRHa in FET can enhance the chances of successful clinical pregnancy, live birth, and implantation, particularly in individuals who have experienced multiple failed implantation attempts (26). A study also showed that there were no statistically significant differences in clinical pregnancy, live birth, abortion, multiple pregnancy, and biochemical pregnancy rates in patients with endometriosis between the two protocols (12). Our results showed that the GnRHa-HRT cycle could improve clinical pregnancy, live birth rate, and embryo implantations, although there were no statistically significant differences in the rate of live birth. In addition, the biochemical pregnancy and early abortion rates of GnRHa-HRT are significantly higher than HRT, but the sample size of miscarriages was small. Overall, GnRHa-HRT showed better clinical outcomes.

Multifactorial logistic regression analysis showed that the clinical pregnancy rate was significantly higher in the GnRHa-HRT group than in the HRT group although there was no significant difference in the live birth rate. As successful embryo implantation is also important for clinical outcomes, we also performed further analysis of embryo implantation, and the results showed that the implantation rate was higher in the GnRHa-HRT group. Although there have been previous studies comparing GnRHa-HRT and HRT, there is a lack of stratified analysis of embryo transfer types for the two protocols. Some studies have shown that transferring embryos at the blastocyst stage leads to a higher pregnancy success rate compared to transferring embryos at the cleavage stage (27, 28). To avoid the impact of differences in embryo type transferred on clinical outcomes, this study compared the outcomes of transferring blastocyst-stage and cleavage-stage embryos. We found that patients who received blastocyst-stage embryos, not cleavage-stage embryos, had significantly higher rates of clinical pregnancy and embryo implantation in the GnRHa-HRT cycle group compared to the HRT group, although there was no significant difference in the live birth rate.

Previous studies had inconsistent conclusions about the two protocols, which may stem from differences in study populations and methodology between different research teams, and the limitation of the sample size is also an influencing factor. Our study had a broader inclusion, was not limited to a single cause of disease, and had a relatively large sample size, further providing confirmation and supplement for favorable clinical outcomes in the GnRHa-HRT cycle. In previous studies, GnRHa demonstrated a notable ability to decrease the inflammatory response and formation of new blood vessels in women with endometriosis, uterine myoma, and adenomyosis (29). GnRHa may have a direct immunomodulatory effect by disrupting the imbalance between Th17 and Treg cells, thereby enhancing endometrial receptivity (30). In addition, it can enhance endometrial receptivity by enhancing the expression of αγβ3 integrin in the endometrium or through IL-6 and IL-11 expression levels of endometrial stromal cells regulated by the miR-124-3p, and increase the number of pinopodes to favor embryo implantation (31, 32). These may account for the favorable clinical outcomes of the GnRHa-HRT protocol.

Meanwhile, as a retrospective study, this study also has some limitations. Since the protocols are decided by physicians and based on patient characteristics, differences in initial parameters between these two groups are inevitable. A significant difference in the mean EMT between the two groups was observed (9.6 mm vs. 10 mm). However, this difference was considered clinically non-significant, as most previous studies have defined an EMT of more than 8 mm as ideal. Second, some confounding factors may inevitably affect the statistical analysis results, although the confounding factors were adjusted, and stratified analysis was performed based on the type of embryo transferred. Furthermore, additional future studies that are prospective and randomized are necessary to confirm the optimal protocol for FET cycles.

## 5 Conclusion

In FET cycles, leuprorelin (as a GnRHa) downregulation combined with HRT may be effective in improving the clinical outcome of patients compared to the HRT cycle. This combination may be more beneficial for patients undergoing blastocyst transfer as it can increase the chances of clinical pregnancy and successful embryo implantation.

## Data availability statement

The raw data supporting the conclusions of this article will be made available by the authors, without undue reservation.

## Ethics statement

The studies involving humans were approved by the Ethics Committee of Henan Provincial People’s Hospital. The studies were conducted in accordance with the local legislation and institutional requirements. The participants provided their written informed consent to participate in this study.

## Author contributions

HH: Data curation, Formal analysis, Investigation, Software, Writing – original draft. ML: Data curation, Investigation, Writing – original draft. CZ: Data curation, Methodology, Supervision, Validation, Writing – original draft. SZ: Data curation, Methodology, Supervision, Validation, Writing – original draft, Formal analysis, Investigation, Writing – review & editing.
